# Supplementary material for: Getting More Out of Biomedical Documents with GATE's Full Lifecycle Open Source Text Analytics
Source: PLoS Comput Biol. 2013 Feb 7;9(2):e1002854. doi: 10.1371/journal.pcbi.1002854 (PMC3567135; doi:10.1371/journal.pcbi.1002854)
Supplement: Dataset S1 — GATE software. Dataset S1 bundled with this paper contains a distribution of GATE (or see http://gate.ac.uk/download/). (TGZ) [file pcbi.1002854.s001.tgz › plos-gate/plugins/Gazetteer_LKB/doc/mail-lists.html]

The Large KB gazetteer - Project Mailing Lists


---

Last Published: 2009-10-17

---

##### Large KB Gazetteer

- Overview
- Usage
- Q & A
- GATE Compatibility
- GATE Gazetteer Feature Comparison
- Semantic Enrichment PR
- Samples

##### Developer Documentation

- Dictionary Lifecycle

##### Project Documentation

- Project Information
  - Continuous Integration
  - Dependencies
  - Dependency Management
  - Issue Tracking
  - **Mailing Lists**
  - Plugin Management
  - Project License
  - Project Plugins
  - Project Summary
  - Project Team
  - Source Repository
- Project Reports

## Project Mailing Lists

These are the mailing lists that have been established for this project. For each list, there is a subscribe, unsubscribe, and an archive link.

| Name | Subscribe | Unsubscribe | Post | Archive |
| --- | --- | --- | --- | --- |
| KIM Discussion | Subscribe | Unsubscribe | kim-discussion@ontotext.com. | ontotext.com |

---

©
2009
Ontotext AD

---
